# Stigma and Time: A Longitudinal Qualitative Analysis of Co-occurring HIV and Tuberculosis Stigma in South Africa

**DOI:** 10.1007/s10461-025-04803-x

**Published:** 2025-07-16

**Authors:** Alanna J. Bergman, Michael V. Relf, Nomusa Mthinkhulu, Nkateko Ndlouvu, Kelly Lowensen, Jason E. Farley

**Affiliations:** 1 University of Virginia, School of Nursing, Charlottesville, VA USA; 2 Johns Hopkins University Center for Infectious Disease and Nursing Innovation, Baltimore, MD USA; 3 Johns Hopkins University School of Nursing, Baltimore, MD USA; 4 Johns Hopkins University Center for Infectious Disease and Nursing Innovation, Port Elizabeth, Republic of South Africa; 5 Duke University School of Nursing, Durham, USA

**Keywords:** HIV, Tuberculosis, TB, Stigma, Qualitative, Longitudinal

## Abstract

For people with HIV and tuberculosis (TB), stigma may change over time. Identifying time points when individuals are most likely to experience HIV or TB related stigma, or when stigma begins to abate, may be useful in tailoring stigma-reduction interventions in resource-limited settings. This study used longitudinal qualitative data to explore if and how HIV and TB stigma change over the course of treatment. People living with HIV and rifampicin-resistant TB were purposively recruited at a district TB hospital in KwaZulu-Natal, South Africa. Participants consented to in-depth interviews throughout TB treatment. The team used reflexive thematic analysis to develop latent themes within the transcripts. This study was designed to identify longitudinal changes stigma over time from the perspective of someone living with HIV and TB. However, participants were more expansive in their conceptualization of evolving stigma. 30 individuals discussed changes in stigma from three distinct perspectives. First was a *perspective of lived experience*, where participants described changes in experienced, internalized, and anticipated stigma over time beginning with diagnosis. The second was from a *shifted perspective*, as participants described their diagnosis and movement from status neutral to status positive transitioning from a potential enactor of stigma to someone at risk for experiencing stigma. Finally, participants described changes in stigma from the *community perspective* whose attitudes towards HIV and TB disease were shaped by time. To strengthen care engagement, we must effectively intervene on disease-related stigma. Appropriate interventions must consider time and shifting social expectations that impact stigma.

**Supplementary Information:**

The online version contains supplementary material available at 10.1007/s10461-025-04803-x.

## Introduction

Despite advances in HIV and tuberculosis (TB) treatment efficacy and tolerability, and reductions in TB treatment duration, South Africa remains significantly burdened by each [1–3]. While biomedical advances are one essential component of HIV/TB elimination efforts, eradication requires consideration of the social factors that drive transmission, and hinder care-seeking and long-term care engagement. HIV/TB testing, prevention, medication adherence, and engagement behaviors are complicated and impacted by a variety of social factors including costs of treatment [4], distance from treatment [5, 6], treatment self-efficacy [7, 8], and psychosocial factors like disease-related stigma [9–11].

Stigma is predicated on dehumanization [12]. Labels and stereotypes highlight differences between groups, these are used to separate one group from another pointing to alleged deficiencies and creating a hierarchy where some groups receive full benefits of social inclusion and others do not [12–14]. Both HIV and TB related stigma are well documented and have been correlated with poorer care engagement behaviors such as hesitancy towards testing [15–17], reduced treatment adherence [18–20], and lower rates of HIV viral suppression [9].

While many studies demonstrate the association between stigma and poorer HIV/TB outcomes, the vast majority of these are cross sectional [21] and cannot evaluate causation. Further, most cross-sectional stigma studies do not consider an individual’s location in their treatment journey and whether location in the care cascade impacts the relationship between stigma and care engagement. This is particularly concerning in lifelong HIV treatment, and in rifampicin-resistant TB (RR-TB), which can last up to 18–24 months.

For people with HIV (PWH) and/or RR-TB who remain in care for extended periods, stigma may fluctuate in response to treatment efficacy, witnessed or experienced prejudice or discrimination, as well as the individual’s knowledge of their disease and their self-efficacy for care engagement and adherence. Each of these may change throughout the course of treatment. Identifying time points when individuals are most likely to experience HIV or TB related stigma, or when stigma begins to subside, may be useful in tailoring stigma-reduction interventions. This study used longitudinal qualitative data to explore if and how HIV and TB stigma change over the course of treatment.

## Methods

This research was conducted at a district hospital in KwaZulu-Natal, South Africa which provides in-patient and outpatient HIV and RR-TB services to peri-urban and rural communities. Enrollment began in March 2022, and straddled the South African roll out of the bedaquline, pretominid, linazolid regimen for drug-resistant tuberculosis. Thus, this analysis includes participants on both long- and short-course all oral regimens. Longitudinal stigma aims were nested within a randomized control trial measuring the efficacy of an escalating m-health intervention aimed at improving HIV and RR-TB treatment adherence. The parent study did not have aims related to stigma or stigma reduction but participants did receive general education about HIV and TB including principles of U = U or “undetectable equals untransmittable”, and similar messaging about the transmissibility of TB following treatment initiation and culture conversion. Eligibility criteria for the parent study included adults 18 years or older living with HIV, GeneXpert positive for RR-TB (including multi-drug resistant, fluroquinolone-resistant and extensively drug-resistant TB), and reported literacy in one of four South African national languages (Afrikaans, English, isiXhosa and isiZulu). Participants assessed as stable for outpatient treatment by a medical officer were invited to participate. Only people diagnosed with both HIV and RR-TB were eligible for the parent study.

Participants from the parent study were invited and additionally consented to participate in longitudinal qualitative interviews throughout RR-TB treatment. In addition to the parent study inclusion and exclusion criteria, the qualitative sub-study required conversational fluency in either English or isiZulu. Interviews were conducted sequentially at entry into RR-TB care (baseline), at month three, and between months six and nine of RR-TB treatment between March 2022 and November 2023. To further contextualize the qualitative findings, the team abstracted demographic and clinical data from the parent study.

Demographic information was collected on the day of study enrollment and entered directly into Research Electronic Data Capture (REDCap). Gender, age, employment, income, and preferred language were collected via participant self-report. Income was dichotomized based on the South African upper bound limit of poverty in 2021. At that time, a monthly income of R1,335 was the upper threshold of poverty equivalent to approximately $90.37 per month [22]. Body mass index (BMI), TB treatment history, and time since HIV diagnosis were abstracted from the medical record. HIV viral load was collected via the parent study and results were retrieved from the South African National Health Laboratory Service. Quantitative data was explored descriptively and used to illustrate the sample to provide context and evaluate transferability.

The team elected to purposively enroll qualitative participants to maximize variability. The initial ten participants were enrolled consecutively. Additional participants were approached to increase variability on sex, age, and time since HIV diagnosis. Two Black women (AJB and NN) trained in qualitative interviewing techniques, conducted the in-depth interviews. Interviews ranged from 35 to 75 min, the average baseline interview was 67 min, and subsequent interviews were generally shorter. The interview guide explored perceptions of self-concept and identity, HIV and TB-related stigma, care engagement behaviors, and mechanisms for coping with stigma. The guide was translated into isiZulu and reviewed by local research assistants for clarity and cultural relevance. Interviews were recorded, transcribed verbatim, and translated to English by a trained local research assistant before uploading into Atlas.ti for coding. During follow up interviews, we reviewed concepts and quotes from the prior interview to allow participants to clarify meaning and respond to preliminary analysis.

The team used reflexive thematic analysis to identify latent concepts within the interviews and develop themes [23, 24]. The primary investigator developed a flexible coding schema which allowed for application of a priori codes from stigma theory and seminal research related to HIV and TB stigma [13, 25–28]. The team evaluated codes for appropriateness and abandoned a priori codes that were not reflected with the data. Inductive codes were added as new concepts and ideas arose. The analytic team was attuned to evolution in code meanings or shifts between or across participants and time consistent with trajectory analysis [29, 30]. Two researchers, one US-based and the second South African, straddled emic and etic perspectives enhancing participant comfort, encouraging reflexivity, and strengthening the credibility of interpretation [31].

### Ethics

The study protocol was approved by the Johns Hopkins University School of Medicine internal review board (approval # IRB00211518) and the University of the Witwatersrand Human Research Ethics Committee (M190937). All participants provided written informed consent to participate in the parent study and in the qualitative sub-study.

## Results

Thirty people living with HIV and RR-TB participated in at least two interviews. Of those, 24 (80%) completed all three interviews with six participants either withdrawing from the parent study or transferring care to another hospital. Participants were 59.3% male, primarily isiZulu speaking (86.4%), with a mean age of 38.5. Most participants were unemployed (59.3%) and living below the South African poverty line (less than R1,335 per month [22]) (55.9%). More than half had been treated previously for TB (62.7%), and on average had been living with HIV for 6.4 years. Upon entry into the study 50.8% had a laboratory confirmed HIV viral load ≥ 200. Overall participant demographics and characteristics by baseline HIV viral load are listed in Table 1.

**Table 1:** Participant demographics

Characteristic	Total	HIVVL < 200	HIVVL ≥ 200	*P* value
Sample size	30	15	15	
Parent study arm				
CHW adherence support	12	5	7	0.456
Standard of care	18	10	8	
TB Treatment history				
Prior TB treatment	20	9	11	0.439
No Prior treatment	10	6	4	
Sex				
Male	19	7	12	0.058
Female	11	8	3	
Employment				
Unemployed	17	7	10	0.532
Full-time employment	10	6	4	
Part-time employment	3	2	1	
Income				
Income < R1,335/ month	12	8	4	0.136
Income ≥ R1,335/ month	18	7	11	
Language				
isiZulu	25	10	15	**0.05**
isiXhosa	4	4	0	
English	1	1	0	
Age in years	36.6	31.6	41.6	**0.003**
Weight in kilograms	57.4	60.4	55.5	0.245
Time since HIV diagnosis	6.15	5.7	6.5	0.756

Bold values indicate statistical significance at *p* < 0.05

This study was designed to identify longitudinal changes stigma over time from the perspective of someone living with HIV and RR-TB. However, participants were much more expansive in their conceptualization of how stigma evolved over time. Participants discussed changes in stigma from three distinct perspectives. The first was a **perspective of lived experience**, where participants described changes in experienced, internalized, and anticipated stigma over time beginning with diagnosis. This theme was built across interviews from one session to the next. The second was from a **shifted perspective**, as participants described their diagnosis and movement from status neutral to status positive which corresponded with a transition from a potential enactor of stigma to someone at risk for experiencing stigma. Finally, participants described changes in stigma from the **community perspective** whose attitudes towards HIV and TB disease were shaped by time. Themes two and three were informed by reflections back in time, sometimes seen in the longitudinal interviews, but other times recalled feelings and experiences from years past that did not occur during RR-TB treatment.

### Theme (1): Changes in Stigma from a Lived Perspective

Overall, participants described a reduction in stigma as treatment progressed. This was first noted between the baseline and month three interviews, and then confirmed between interviews at months three and six. Most participants endorsed some form of internalized or anticipated stigma at baseline, and approximately half reported experiencing stigma in the form of isolation, gossip, rejection, or discrimination. At baseline, stigma associated with TB was largely attributed to the characteristic cough and weight loss which fueled community fears of infection. Stigma associated with HIV appeared to incur more internalized stigma. An HIV diagnosis also carried judgement about responsible conduct and whether society deemed an individual culpable for acquiring HIV through their own “negligence” or “immoral” behavior. Women in particular qualified the circumstances which led to HIV exposure, explaining that they acquired HIV perinatally or from a monogamous and trusted partner in order to minimize judgement from healthcare workers and researchers.

Several participants who experienced TB stigma in the form of social isolation described how over time, friends and family returned as though nothing had changed. Because the same individuals who abandoned them at the beginning of treatment later returned, some participants questioned whether they imagined being stigmatized. At baseline, one participant explained, “*When one is sick*,* people don’t want to be around you and even your friends distance themselves from you. It is a painful thing*”. During his next interview he was unsure, “*At first*,* I experienced stigma*,* or maybe I was judging myself because I would see how people do or say things and I would conclude that they are referring to me and maybe I was wrong. Maybe it was me who had a problem*” (31-year-old Male, Month 3). As this participant saw his friends return, he began to negate his experience. He turned inward towards self-doubt, exemplifying the insidious and demoralizing nature of social stigma.

By month three, many participants saw clinical improvements including increased stamina and weight gain. This physical improvement reduced illness visibility allowing identity concealment and lessened stigma as treatment progressed. Longitudinal data from participants who exemplified changes in stigma over time accompanied by relevant clinical data such as weight, clinical symptoms, and viral load are available in Supplemental File 1.

Many participants who experienced HIV or TB stigma attributed their reintegration into social life to a return to health. One man noted, *“My friends used to isolate me before I started treatment because they knew that TB is a transmittable disease*,* but since I started treatment*,* they are fine*,* and they no longer isolate me…I think because they see now that I am getting better since I am taking my treatment and I am no longer coughing”* (42-year-old male, Zulu, Month 3).

At baseline, many participants anticipated stigma due to weight loss, reductions in strength, and hyperpigmentation resulting from clofazimine exposure during RR-TB treatment. Physical signs provided a visual clue, and participants anticipated that others would identify them as someone with TB or HIV, based on their physical presentation. A male participant shared how changes in his appearance led to anticipated stigma. *“Ok*,* when I was first diagnosed* [with HIV], *I used to feel like that…like I hated myself. I used to stay indoors*,* scared of going outside because I thought people will see that I am sick and talk about me* [anticipated stigma], *but eventually I got over it”* (38-year-old male, Zulu, Month 6). Over time he adjusted and acknowledged that others could not see his HIV status. This evolution in thought coincided with improvements in symptoms and outward signs. When the same participant was interviewed for a second time, he had gained 9 kg and had full resolution of cough, dyspnea and pleuritic chest pain which may have reduced his fears of being seen by others.

Physical manifestations also changed participant self-perception even if others in the community could not see outward changes. A female participant explained how she saw changes in her weight that others did not notice,

*“I am the one that’s saying I have lost weight. At home they say ‘No you haven’t’*,* and I say ‘No*,* I know myself*,* I am losing weight’…Yes*,* I can wear my clothes*,* but I know myself*,* I am losing. Even my doctor*,* I went to my doctor last week and he said*,* ‘Hayibo! You are looking fresh*,* your face is full!’ and I’m like doc*,* don’t patronize me*,* because I don’t feel ok’”* (35-year-old female, Zulu, Month 3).

This participant experienced reduced self-concept stemming from weight loss. She did not attribute the loss exclusively to HIV *or* RR-TB, rather she blamed both HIV *and* RR-TB for changing her appearance. It was not until her final interview that she began to feel like herself again; after nine months of RR-TB treatment she gained 7 kg finally achieving her desired appearance which camouflaged her diagnoses.

Participants also drew a link between treatment and a return to health. They saw how antiretroviral therapy and TB treatment quickly moved them towards health. In this way, treatment was an indirect intervention to reduce stigma.

### Theme (2): Changes in Stigma from a Shifted Perspective

The interview guide prompted participants to reflect on stigma that they internalized, witnessed, anticipated or experienced *after* diagnosis. However, many participants reflected back, prior to the baseline interview, and focused on the shift from pre-diagnosis to diagnosis. Diagnosis was a profound moment for many participants and represented a shift in identity from a “well person” to an “ill person” fundamentally changing how they saw themselves. Coinciding with this change in illness identity, some participants recognized that prior to diagnosis they were enactors of stigma who had unfairly labeled and stereotyped people living with HIV/TB.

Regarding HIV, multiple participants mentioned how they previously believed that PWH were “badly behaved” and deserved their diagnosis due to their sexual behaviors or due to the influence or alcohol or other substances. Participants discussed their prior perception of TB as a frightening disease that required strict isolation and social exclusion to prevent transmission. While most participants lacked a nuanced understanding of the differences between drug-sensitive TB and RR-TB at baseline, there was an overall acknowledgement that RR-TB was worse, and occasionally characterized as “big” TB.

Participants discussed how their diagnoses forced them to self-reflect and dismantle internalized preconceptions about guilt or blame in HIV/TB transmission. *“I think I was a bit judgmental towards them* [PWH] *thinking that only people who are sexually promiscuous get HIV*,* but after I found out that some people like me*,* can get it from their parents*,* I stopped judging”* (21-year-old female, Zulu, month 6). This participant acquired HIV through perinatal contact, her mother died from complications related to HIV. The participant was not tested until she became symptomatic at age 14, prior to her sexual debut. Upon learning her HIV status, she had to dismantle her previously learned stereotypes and rebuild her mental archetype of someone with HIV.

In several cases, interviews were an opportunity for internal reflection. Participants had time to process previously unacknowledged social and psychological shifts. Introspection allowed participants to feel empathy for others living with HIV/TB, and to feel empathy for themselves. One participant denied experiencing or internalized TB stigma himself but did detail prior misconceptions and a new outlook, *“I don’t think of TB like I used to anymore. I now feel sorry for people who have TB because I now understand TB*,* especially those who are not on treatment yet”* (35-year-old male, Xhosa, Month 6).

Participants acknowledged a socialized tolerance of infectious disease stigma which informed biases towards HIV/TB. Participants’ individual experiences with HIV and TB humanized each disease. Diagnosis often compelled an unraveling of unconscious perceptions of people with TB and HIV.

Many changes in empathy resulted from increased infectious disease knowledge. Information about treatment to either suppress HIV or cure TB was particularly influential, *“I think my thoughts have changed*,* because now I have experienced it myself. I think TB is not dangerous when you take treatment and there is no need for stigma”* (37-year-old female, Zulu, Month 6). As participants learned about transmission and the impact of treatment on public health, they began to reject the negative labels and fears ascribed by the wider community (See Table 2).

**Table 2:** Evolving empathy corresponding with changes in illness identity

Participant characteristics	Quote related to TB or HIV	Quote reflective a shift from pre-diagnosis to present
Sex: Male Age: 39 8 years since HIV dx Prior TB treatment: Yes Social proximity to HIV: Yes—brother	HIV	[Reflecting back to earlier in the HIV epidemic] “It was scary to be in the same places as a person living with HIV, I think it was as a result of lack of knowledge about HIV…I think it was because we knew that there is no cure or medication for HIV so ultimately the person is going to die”
Age: 36 Sex: Male 6 months since HIV dx Prior TB Treatment: No Social proximity to TB: Yes brother died from RR-TB	TB	“I was scared of it [TB], I would even panic when people cough around me, I wouldn’t show any signs of being afraid but in my mind, I would have bad thoughts. Even when I was diagnosed, I was shocked…My thoughts have changed now because I also have TB now and I have gained knowledge about it”
Sex: Male Age: 34 7 years since HIV dx Prior TB treatment: No Social proximity to HIV: Yes—close friends	HIV	“I honestly had stigma against them [PWH], and I didn’t want to be around them, but now I am fine because I understand HIV”
Sex: Female Age:35 < 6 months since HIV dx Prior TB episodes: No Social proximity to TB: No	TB	“I think my thoughts have changed, because now I have experienced it [TB] myself. I think TB is not dangerous when you take treatment and there is no need for stigma”
Sex: Male Age: 39 9 years since HIV dx Prior TB treatment: No Social proximity to HIV: Yes—close friends	HIV	“I used to know HIV as a dangerous disease, but in a long run, I got infected with HIV and I realized that HIV is not dangerous. If you treat it in a correct manner and adhere to treatment. At first, I was really scared of HIV. I’ve been living with it from 2014 and I have never been sick because of it

*dx*= diagnosis

### Theme (3): Changes in Stigma from the Community Perspective

This study did not set out to evaluate community level stigma, however, as participants discussed their lived experiences with stigma, shifts in community stigma were directly tied to the way that participants anticipated, perceived and internalized stigma. Participants explained how community stigma shifted over time in response to local disease burden, advances in treatment, and in response to health education campaigns.

Surprisingly, several participants in this study felt that between the two, TB carried more stigma in their communities than HIV. Among this sample, the general perception was that over the course of the South African HIV epidemic, the negative labels and stereotypes associated with HIV dissipated. This was due in part to the burden of HIV in South Africa. All participants reported having friends or family living with HIV. HIV was seen as a common occurrence and participants knew the individual stories of loved ones who were affected by HIV. *“I will tell you the truth*,* most people are exposed to HIV because most of us are living with HIV. My sisters are on HIV treatment as well*,* so it was easy for them to adjust to my diagnosis”* (36-year-old male, Xhosa). For some, the ubiquity of HIV made it seem as though HIV was a mundane reality of social life. Participants also knew that community perceptions of HIV had changed over the years in response to improvements in antiretroviral therapy and reduced morbidity and mortality. As one man put it, *“At first HIV was a threatening factor*,* but ever since the ARVs* [antiretroviral therapy] *were released for the public*,* it is not a worrying factor anymore”* (49-year-old male, Zulu).

As a result, several participants diagnosed after the advent of highly active HIV treatment explained that they were welcomed into a community of people living with HIV. One man described the adherence support he received from others living with HIV, *“all of us at home are on ARVs*,* so when you see one person drinking their medication*,* even when you had planned to skip it for that day*,* they encourage you not to miss your medication”. (*30-year-old male, Zulu, Month 6*).* All of these contributors led to an overall feeling that TB was a more stigmatized disease because of its transmissibility, perceived infrequency compared to HIV, and lack of familiarity with TB (See Table 3).

**Table 3:** Community desensitization towards HIV

Participant characteristics	Changes in community perspectives towards HIV
Sex: Female Age: 25 Time since HIV dx: < 6 months Social proximity to HIV: Yes—mother	“I think most people who have stigma against HIV are older people. Most [younger] people have knowledge of HIV now”
Sex: Female Age: 25 Time since HIV dx: 6 years Social proximity to HIV: Yes—friends	“Yes, I used to have that worry [about stigma] when I was young because stigma is an old thing, but growing up and seeing that a lot of people are living positively with HIV, I don’t see stigma anymore”
Sex: Male Age: 47 Time since HIV dx: < 6 months Social proximity to HIV: Yes—friends	“I think people used to experience [HIV] stigma in the olden days, but now people are educated about HIV, and they understand…They do not have stigma [towards HIV]”
Sex: Female Age: 22 Time since HIV dx: < 6 months Social proximity to HIV: Yes—friends & extended family	“I think TB carries most stigma because people are used to HIV now and it is not as scary as it used to be”
Sex: Female Age: 32 Time since HIV dx: 7 years Social proximity to HIV: Yes—sisters	“Like I said now, in our days HIV is just like Flu, maybe they used to have stigma back in the days, but now, there is no stigma against people living with HIV. I think there is still stigma associated with TB because people know it is a transmittable disease and they are still scared of it”

## Discussion

In this study, people living with HIV and RR-TB described changes in stigma over time from three perspectives not previously described in the stigma literature. Individuals living with infectious diseases have the potential for long term management and/or cure. As people are diagnosed, engaged in care, and started on treatment they will likely reconsider who they are within the context of their diagnosis and where they fit into a social scheme. While someone with HIV and/or TB is at risk for stigma, their relationship with stigma is constantly shifting in response to individual and social factors.

Though long neglected, time is an essential component in evaluating and intervening on stigma [32]. Longitudinal analyses of stigma are relatively new and are poised to help explore the shifts in stigma that occur as individuals move in and out of illness identities across diagnosis, chronic management, and cure. As individuals achieve durable viral suppression on antiretroviral therapy and achieve TB treatment completion and cure, their illness identity may change, impacting internalized stigma and changing their risk for experienced stigma.

Some HIV stigma literature indicates that internalized HIV stigma is more strongly associated with HIV outcomes than other types of stigma such as experienced or perceived community stigma [9, 10]. Less is known about TB stigma and its associated engagement outcomes. At this time there are no TB stigma instruments with established predictive validity towards TB outcomes, nor are there TB stigma instruments validated on their temporal stability across the care cascade [21]. Further, the specific overlap that occurs due to the intersections of HIV, TB and their stigmas is understudied. Given that the TB epidemic in South Africa is driven in part by HIV [1], the specific components of intersectional stigma and their impact on care engagement are of high interest [27].

Among our participants who were dually burdened by TB and HIV, physical appearance and visual cues towards illness had an impact on lived stigma. This is consistent with prior literature and stigma theory which suggests that illness visibility or potential for concealing a stigmatized identity directly impacts the type and degree of stigma that an individual encounters [12, 25, 26]. ART and antitubercular medications can be a powerful tool in mitigating lived stigma and may be useful in promoting adherence. However, addressing stigma directly should be focused on the community at large as the primary enactors upholding social stigma.

Many of the shifts noted by participants occurred after exposure to someone living with HIV or RR-TB. This data is consistent with theoretical literature. Contact theory asserts that prejudice and stereotypes are mitigated by contact or proximity between groups [33]. Analysis of contact theory has demonstrated three key facets of stigma reduction through direct contact: knowledge, anxiety reduction, and empathy [33]. Increased knowledge about the minority or stigmatized group may help to dismantle negative and biased stereotypes reducing stigma. In HIV, learning about people living with the virus, modes of transmission and important public health messaging such as “undetectable equals untransmittable” or U = U may help to alleviate perceived threats such as the risk of casual transmission. Similar messaging about medication adherence and reduced transmission may dismantle fears of TB infection.

In infectious disease education, knowledge and anxiety reduction go hand in hand. Through knowledge and contact with a stigmatized group, anxiety about transmission, safety, and personal risk may be assuaged. Prior studies have established that much of the stigma surrounding infectious diseases is related to fears about infection and personal safety [34] and that accurate disease-related knowledge is correlated with lower levels of stigma [15, 35]. From this analysis, direct contact with a stigmatized group appears to increase empathy. As members of the majority group, (in this case undiagnosed majority without TB or HIV) have repeated contact with a stigmatized group, majority individuals may begin to appreciate between group similarities, in effect, re-humanizing the stigmatized minority.

Contact theory provides a reasonable explanation for the longitudinal changes in stigma noted by participants in this study. Contact exposure would explain increased empathy following diagnosis and reduction in community stigma over time. Additionally, contact theory may explain improved self-concept over time as individuals interact with others living with HIV and TB in care settings, and in the larger community. This is well demonstrated in HIV literature where people who have direct contact with someone living with HIV are less likely to endorse stigmatizing views and less frequently support discriminatory policies [36]. In another recent study, researchers established a significant correlation between prior TB treatment and reduced TB stigma among community members. South Africa has an estimated HIV rate of 17.8% which varies across provinces [37]. With high HIV case rates, social contact to HIV increases. It is unsurprising that perceptions of HIV would shift among the larger status neutral community.

Although there is wide recognition of TB related stigma and its negative impacts, there is far less research on TB stigma to date than HIV stigma. Although TB remains a leading cause of death of PWH [1], TB generally receives less attention and funding than HIV and other communicable diseases [38, 39]. Disparities in funding and public health initiatives may have contributed to the perception among participants that RR-TB was uncommon or mysterious. Indirect contact with people living with TB through social media campaigns and public health initiatives may help to reduce community fears of TB. The HIV epidemic is relatively short compared to the lengthy history of TB. Though concerted efforts, community perceptions towards TB could shift, particularly in a country like South Africa which has a strong public health system and high disease burden.

## Conclusion

This study demonstrated three key areas of shifting stigma: changes in lived stigma over time, changing illness identity and evolving empathy, and community desensitization towards HIV. Participants were clear that infectious disease stigma is not static but in constant flux among those affected, and in the wider community. A return to health via treatment was a driver of shifting stigma among people with lived experience of HIV or RR-TB. Contact with people living with HIV and TB was central to shifts among a status neutral community, normalizing treatment for infectious diseases and humanizing the people living with HIV and RR-TB. To strengthen care engagement, scientists and clinicians must effectively intervene on disease related stigma. Appropriate interventions must consider time and shifting social expectations to keep up with current trends in stigma and adapt to challenges as they arise.

## Electronic Supplementary Material

Below is the link to the electronic supplementary material.

Supplementary Material 1

## Data Availability

Data cannot be shared publicly because it contains protected health information, and due to IRB confidentiality and data sharing restrictions; however, data can be made available for researchers who meet the criteria for access to confidential data. Please contact the Center Manager for The Johns Hopkins Center for Infectious Disease and Nursing Innovation, Kelly Lowensen (klowens1@jhu.edu), for additional assistance accessing study data.
