# Supplementary material for: Stigma and Time: A Longitudinal Qualitative Analysis of Co-occurring HIV and Tuberculosis Stigma in South Africa
Source: AIDS Behav. 2025 Jul 16;29(11):3608–16. doi: 10.1007/s10461-025-04803-x (PMC12500733; doi:10.1007/s10461-025-04803-x)
Supplement: Supplementary file 1 — Supplementary Material 1 [file 10461_2025_4803_MOESM1_ESM.docx]

Changes in Stigma Over Time with Accompanying Clinical Data

| **Participant Demographics** | **Quote related to TB or HIV** | **Baseline quote** | **Follow up quote** |
| --- | --- | --- | --- |
| Age: 35  Sex: Male  Prior TB Treatment: Yes  2 years since HIV dx | TB | "When one is sick, people don’t want to be around you and even your friends’ distance themselves from you. It is a painful thing, but we move on with life... we used to hang out together before I got sick. And it was nice because I had a job then. So, since I got sick, some of my friends changed and stopped being friends with me." | "Yes, there is a change, for example, at first, I didn’t want people to see me or even talk to people because I had lost a lot of weight.  But now I am OK and I am proud of myself, and I can see that I am gaining my weight back." |
| Clinical presentation | | **Baseline**: 45kg; HIV viral load = 620; TB culture positive; s/s none | **Month 6**: 51kg; HIV viral load < 50; no TB culture data available; s/s none; AEs: hyperpigmentation, insomnia |
| Age: 42  Sex: Male  Prior TB treatment: Yes  11 years since HIV dx | TB | “I used to have friends, but they no longer come visit me. I think TB [carries more stigma] because my friends know about [my] HIV [status], but they started distancing themselves when they found out about TB.” | “My friends used to isolate me before I started treatment because they knew that TB is a transmittable disease, but since I started treatment, they are fine, and they no longer isolate me…I think because they see now that I am getting better since I am taking my treatment and I am no longer coughing like I used to before I was on treatment.” |
| Clinical presentation | | **Baseline**: 54kg; HIV viral load = 50; TB culture positive; s/s: chest pain, cough, insomnia; night sweats; weight loss | **Month 3**: 62.8kg; HIV viral load <20; TB culture negative; s/s cough; AEs: hyperpigmentation, neuropathy |
| Age: 31  Sex: Female  Prior TB treatment: No  9 years since HIV dx | HIV | “I have low self-esteem now, and I always feel like everywhere I am people can tell that I am sick.” | [Discussing her decision to disclose her HIV status to others] "I don't want to suffer alone with thoughts, so I find it helpful to talk, and luckily I haven't found people who have stigma." |
| Clinical presentation | | **Baseline**: 64.6kg; HIV viral load = 62; No TB culture data available; s/s cough | **Month 3**: 61.9kg; HIV viral load <50; TB culture negative; s/s weight loss; AEs: none |
| Age: 21  Sex: Female  Prior TB treatment: Yes  6 years since HIV dx | HIV | “For people with HIV, I think people look at them and think they will die soon. I think that is why [there is stigma], they think because you have TB then it means you are going to die." | “At first I used to think that people can see that I am different from them and that I am sick, but as time went by , I realized that it was just my thinking…no one can tell that I am living with HIV.” |
| Clinical presentation | | **Baseline:** HIV viral load = 50; 45kg; TB culture positive; s/s cough dyspnea, fatigue, weight loss | **Month 6:** HIV viral load <50 ;47kg; TB culture negative; TB symptoms resolved; AEs: hyperpigmentation, neuropathy |
| Age: 49  Sex: Male  Prior TB treatment: Yes  16 years since HIV dx | TB | [Following his TB diagnosis], "My self-esteem went down. It is not an easy thing knowing that you have a disease in you." | "A lot has changed [since the last interview]. I have regained myself-esteem because by the time I was told I have MDR-TB I felt that I can't take it anymore. As I mentioned the last time, this is not the first time I am taking MDR-TB treatment and I know how hard it is to take it but I have regained a lot of things. Spiritually and mentally I am ok. Physically I look very good. I am motivated and my family and colleagues are also supportive. Even the community looks at a person who is well when they look at me. " |
| Clinical presentation | | **Baseline:** 87kg; HIV viral load = 8,361; culture negative; s/s chest pain, cough, dyspnea, nausea, night sweats, fatigue, | **Month 3:** 91kg; HIV viral load =36; culture negative; s/s chest pain all other symptoms resolved; AEs: bruising; pruritus; rash, arthralgia, myalgia |
| Age: 34  Sex: Female  Prior TB treatment: Yes  9 years since HIV dx | HIV | "At first I thought that I let my mom down, I let myself down… I was ashamed. I was afraid that maybe somehow, somewhere along the way I didn't take my parents' advice, I didn't act right." | "I'm not feeling any shame now. I was at first…I was so angry with myself….But now I've accepted it and I'm living. Even with this disease we *live*." |
| Clinical presentation | | **Baseline:** 52 kg; HIV viral load = 242,920; TB culture positive; s/s chest pain, night sweats, weight loss, cough, dyspnea, constipation | **Month 6:** 53kg; HIV VL = 32; TB culture negative; TB symptoms resolved; AEs: none |
| Legend: Clinical presentation data corresponds with the first identified change in stigma from the qualitative data.  Dx = diagnosis; s/s = signs and symptoms of TB or HIV; AEs = adverse events associated with HIV or TB treatment | | | |
